# *Mycobacterium tuberculosis* Essential Gene Thymidylate Synthase Is Involved in Immune Modulation and Survival inside the Host

**DOI:** 10.1021/acsomega.4c02919

**Published:** 2024-07-23

**Authors:** Sana Tanweer, Tarina Sharma, Abhinav Grover, Meetu Agarwal, Sonam Grover

**Affiliations:** † Department of Molecular Medicine, Jamia Hamdard, New Delhi-110065, India; ‡ New Jersey Medical School, Rutgers, The State University of New Jersey, Newark, New Jersey 07103, United States; § School of Biotechnology, Jawaharlal University, New Delhi-110069, India

## Abstract

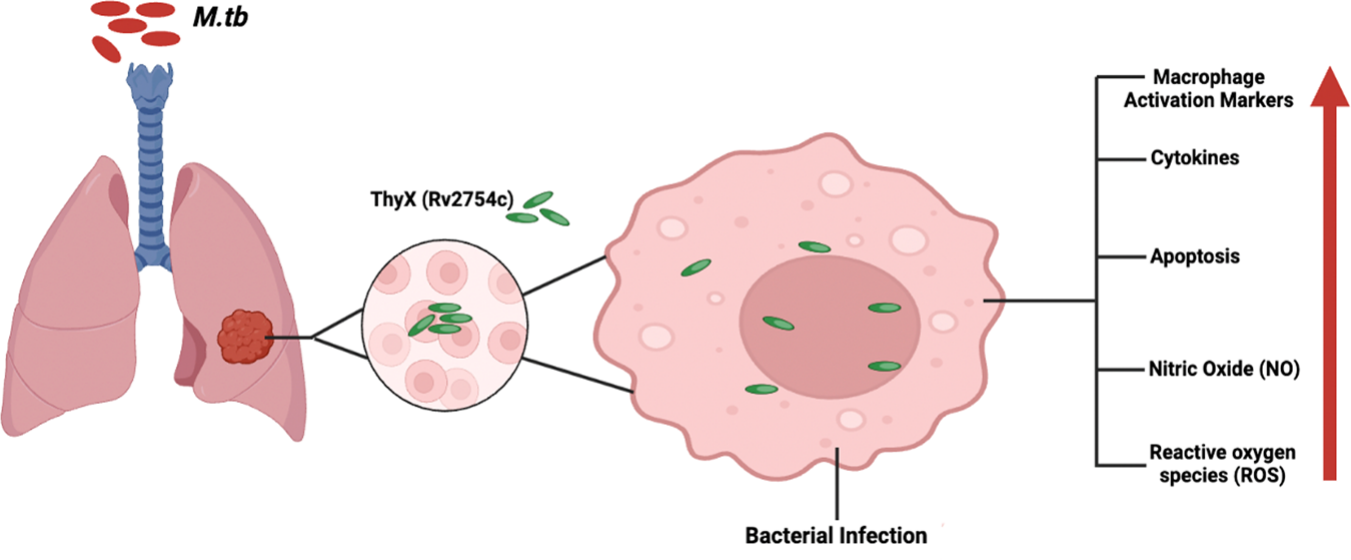

A *Mycobacterium tuberculosis* essential gene, ThyX (Rv2754c), plays a key role in intermediate metabolism and respiration by catalyzing the formation of dTMP and tetrahydrofolate from dUMP and methylenetetrahydrofolate. ThyX is present in the *M.tb* complex and in *M. smegmatis* a nonpathogenic strain of Mycobacteria. In this study, we identified a novel function of ThyX, an enzyme with immune-modulating properties. We have shown that ThyX can activate the macrophages in the host toward M1 response. Overexpression of ThyX stimulates the production of nitrite oxide (NO) and induces apoptosis in macrophages; indeed both responses help the host to control growth of *M.tb*. ThyX was also discovered to play a role in the recombinant bacterium’s ability to survive when it was subjected to oxidative and hypoxic stress by macrophages. These findings demonstrate the protein’s functional importance in *M.tb*. Indeed these findings represent ThyX as a potential candidate for future research and show this as a therapeutic target.

## Introduction

Nearly one-third of the world’s population is infected with tuberculosis (TB), a potentially fatal illness caused by *Mycobacterium tuberculosis* (*M.tb*).^1,2^ A quarter of the world’s population has latent TB infection.^3^ Over the past two decades, the situation has gotten worse due to the emergence of strains that are multidrug-resistant (MDR) and extensively drug-resistant (XDR). With 450,000 incident cases of rifampicin-resistant tuberculosis recorded in 2021, the burden of drug-resistant tuberculosis also increased by 3% between 2020 and 2021. The largest percentages (>50%) of MDR or rifampicin-resistant tuberculosis were found in Russia and other eastern European and Central Asian nations.^2^ Rapid drug resistance strain development has brought attention to the need to investigate *M.tb* virulence mechanisms that have enabled this bacteria to evolve as one of the most effective pathogens known to humans. The ability of *M.tb*, an intracellular pathogen, to survive in host macrophages is a crucial component of its pathogenicity. The intricate strategy used by *M.tb* to thrive in the very microbic environment of macrophages is extremely complex and remains a mystery.

*M.tb* has developed defense mechanisms that let it infect and persist in the host environment while evading the immune system. This necessitates the interaction of many virulence factors that allows *M.tb* to adjust to the host immunological challenges. The slow-growing *M.tb* allowed it to adapt to the lungs by horizontal gene transfer, which boosted its harmful potential.^4−6^

A total of 121 methyltransferases (Mtases) have been found in *M.tb*. H37Rv, which is significantly more than other pathogenic, nonpathogenic, and opportunistic species of mycobacteria.^7^ The methylome of the *M.tb* complex has not been extensively studied. It might be necessary for this pathogen to endure challenging circumstances, including a hypoxic environment, which might increase its virulence and lead to the development of treatment resistance.^8,9^

Recent research has identified a flavin-dependent thymidylate synthase (FDTS) called ThyX as a potential target for the repurposing of existing antibacterial medications.^10,11^*Denovo* 20-deoxythymidine-50-monophosphate (dTMP) synthesis depends on the enzyme thymidylate synthase (ThyA), which is a member of the methyltransferase family. Additionally, ThyX is essential for DNA synthesis because it catalyzes the conversion of dUMP to dTMP, acting as a crucial component for DNA synthesis to continue and, as a result, for cell survival and replication.^12^ Given that ThyX has only sometimes been identified in eukaryotes and is absent in humans, it is an especially popular target for antibacterial drugs.^13^ In addition to other significant human pathogens, the gene is identified in *Bacillus anthracis, Helicobacter pylori*, and *Mycobacterium*. The ThyX gene has been shown in numerous studies to be essential for bacterial survival, and MDR strains of *M.tb* have been shown to overexpress this gene.^14^

In this work, we looked at the functional role of ThyX of *M.tb*. Examinations were conducted into ThyX’s impact on cellular characteristics, including stress resistance and immunological response. To study the responses of this protein *in vivo*, macrophage surface marker estimation, cytokine ELISA, reactive oxygen species, nitric oxide test, and apoptosis were estimated. The impact of ThyX overexpression on immune modulation and pathogenesis was evaluated *in vivo* by evaluating the gain of functions after insertion into nonpathogenic bacteria, *M. smegmatis*.

Our research sheds important new light on the function of ThyX in host–pathogen interactions during TB pathogenesis.

## 2 Results and Discussion

### 2.1 *In-Silico* Analysis of ThyX and Generation of an Overexpression Strain in *M. smegmatis*

ThyX protein sequence consists of 250 amino acids and has a molecular weight of 27.5 kDa. We have performed *in-silico* analysis of the enzyme using different computational tools. First, the VaxiJen tool was used to predict the antigenic nature of ThyX, meaning it can trigger an immune response in humans (Figure S1.A). The B-cell and T-cell epitope analysis using the IEDB server confirmed ThyX’s immunogenicity and strengthened its role in immune modulation (Figure S1.B, C, and D). Next, by P-BLAST analysis, it was found that ThyX does not show any homology with humans and is rarely seen in eukaryotes; hence ThyX is shown to be a highly preferred target for antibacterial drugs.

Further to characterize ThyX, it was cloned and expressed in a pET28a vector (Figure S2A, B and C), and the recombinant protein so obtained was purified by affinity chromatography (Figure S2.D). We wanted to explore the effects of overexpression of ThyX; hence it was subcloned in the pVV16 expression vector and electroporated in the *M. smegmatis* strain (Figure S2E). Positive overexpressed constructs of *M. smegmatis* harboring His-tagged ThyX (*M.s*_ThyX) or vector control pVV16 (*M.s*_Vc) were cultured for further use.

### 2.2 Overexpression of ThyX in *M. smegmatis* Enhances Bacterial Survival

**Figure 1:**
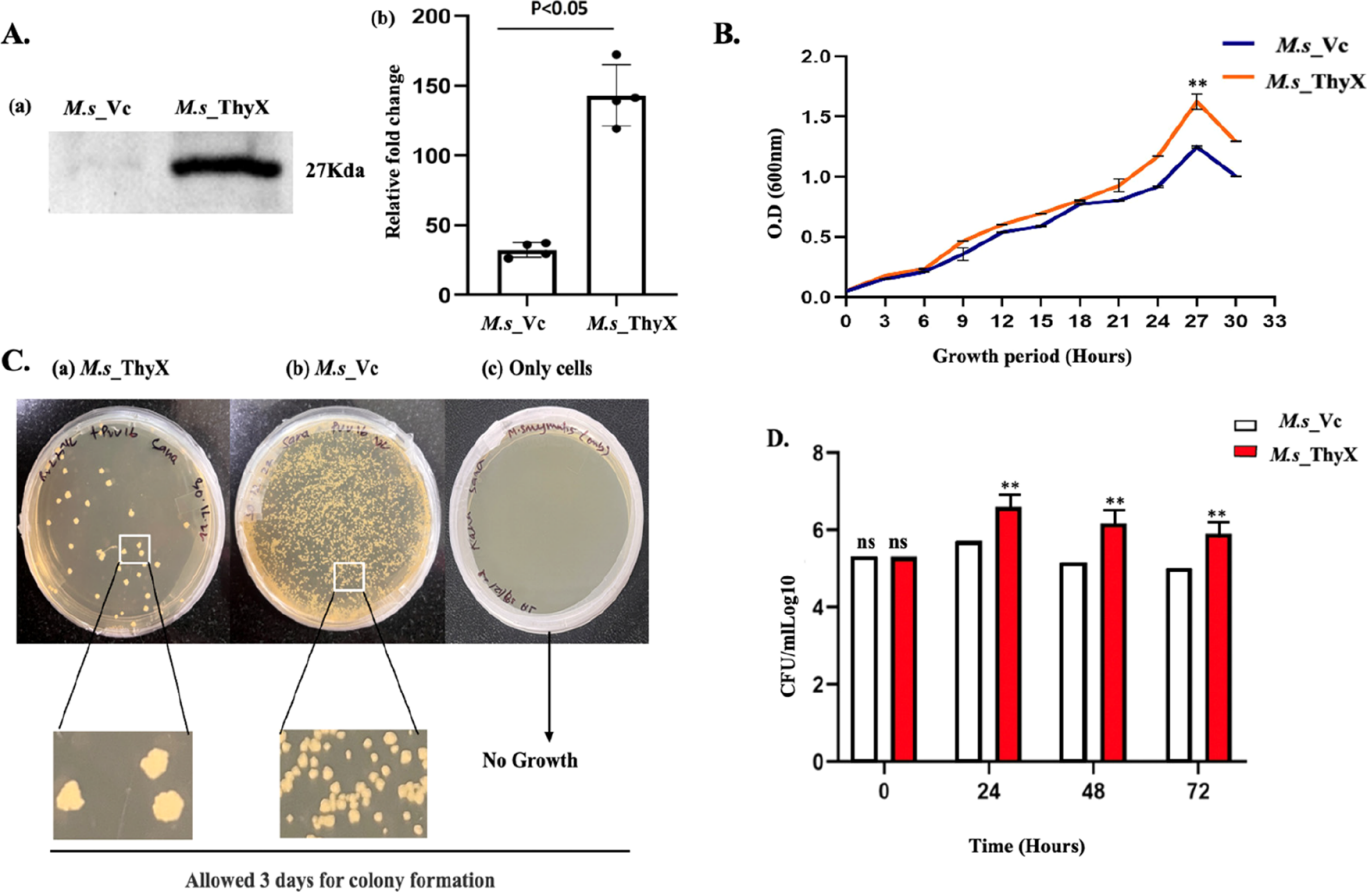
(A) To assess the role of *M.tb* ThyX in pathogenicity, genes from the pathogenic strain of *M.tb* were introduced into the nonpathogenic *M. smegmatis*. (A. a) Confirmation of *M. smegmatis* strain by Western blotting using anti-His antibody. (A. b) Graphical representation of *M.s*_ThyX and *M.s*_Vc using Western blot by the ImageJ tool. (B) Log phase cultures (OD_600_ of 0.8–1.0) of *M. smegmatis* mc^2^ 155 vector control (*M.s*_Vc) and *M.s*_ThyX were diluted 1:100 into 7H9 media and cultured for approximately 12 h until the OD_600_ reached 0.05. Reinoculated cells were then allowed to grow for 30 h, and the surviving cells were grown on LB media after every 3 h in culture. OD_600_ was also taken every 3 h up to 30 h. (C) Plates were inoculated with equivalent amounts of cultures harboring (a) *M.s*_ThyX or (b) *M.s*_Vc from panel B or (c) cells only, at the 30 h time point. Colonies were visible after 3 days. (D) THP-1 cells were incubated with an equal number of *M.s*_Vc and *M.s*_ThyX at an MOI of 1:10 for 4 h. THP-1 cells were lysed at 0, 24, 48, and 72 h postreseeding to extract the intracellular bacteria that survived. Bacteria was plated on LB agar, and the CFU assay was done. For ** the corresponding *P* value is <0.01.

As we have shown in the above result, we have generated overexpression strains *M.s*_ThyX and *M.s*_Vc. First, by doing Western blotting experiments using anti-His antibodies, we confirmed the expression of the ThyX gene in the *M.s*_ThyX strain, and the band of specific size was absent in *M.s*_Vc (Figure 1A.a and A.b). Further, we wanted to see the effects of ThyX overexpression on the growth of bacteria in *in-vitro* conditions; thus we have performed a growth curve analysis. Here we have observed that *M.s*_ThyX grows fast compared to *M.s_Vc*, suggesting that ThyX provides a growth advantage to *M.tb* (Figure 1B). Next, following 30 h of incubation, culture aliquots from the *M.s*_ThyX and *M.s*_Vc were plated to observe colony size and number, and we found that *M.s*_ThyX colonies were larger in size and less in number compared to the higher number of smaller colonies of the *M.s*_Vc (Figure 1C).

Further, we wanted to understand the impact of this overexpression inside the host, and for this, we have used THP-1 macrophages. To do this, *M.s*_ThyX or *M.s*_Vc strains were grown until the mid log phase, and single-cell suspensions were prepared for the infection. Colony-forming unit (CFU) analysis was performed at different time points to see the impact of ThyX overexpression on the survival of bacteria inside the macrophages. Similar to *in-vitro* assays, we have found more CFUs in *M.s*_ThyX-infected cells compared to *M.s_Vc*-infected cells at all time points including 24, 48, and 72 h (Figure 1D). Together these results suggest that ThyX overexpression aids bacterial growth in both *in-vitro* and *ex-vivo* conditions.

### 2.3 *M.s_*ThyX Leads to the Production of NO and Apoptosis in Infected Host Cells

**Figure 2:**
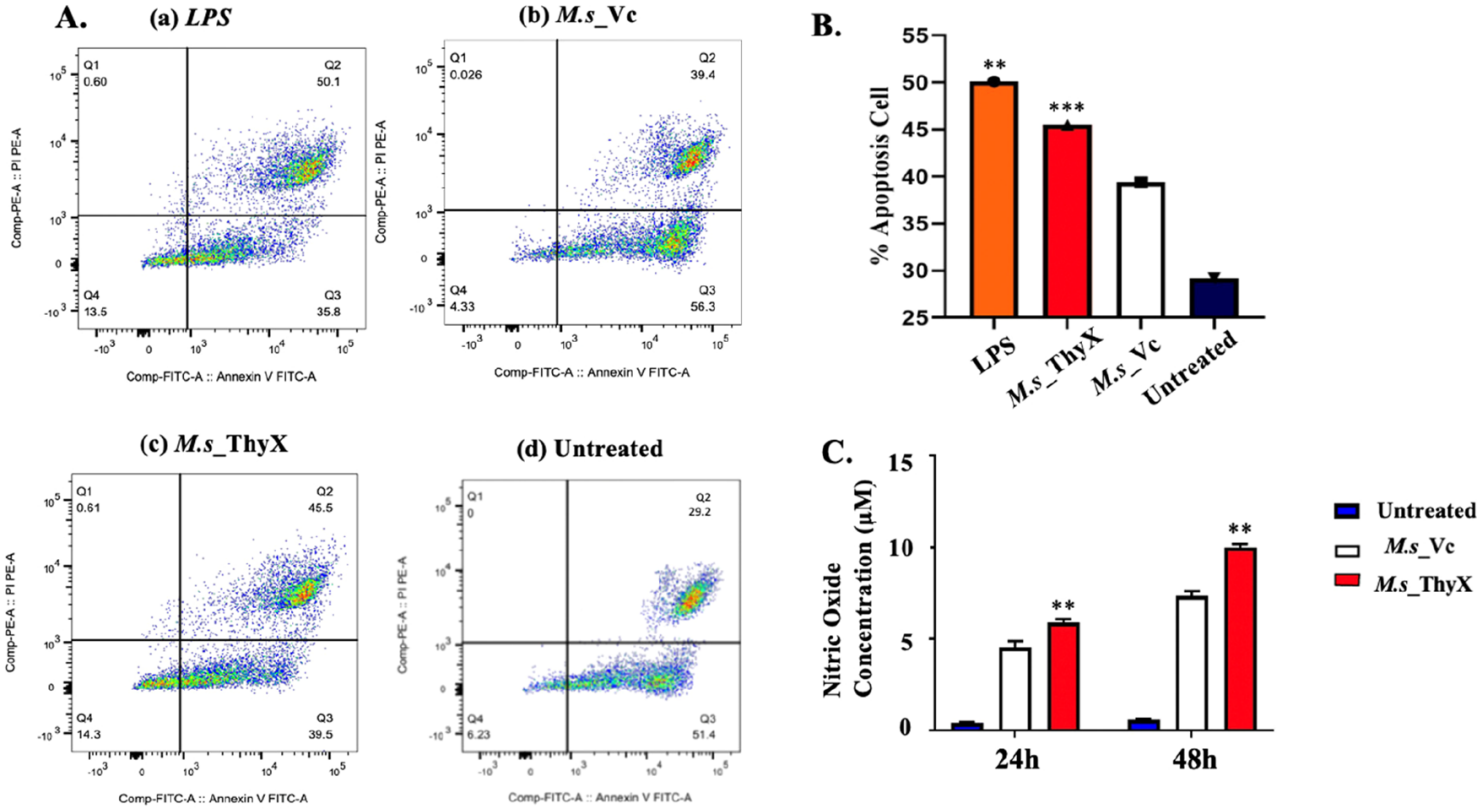
(A) Representative scatter plot of the apoptosis assay at 48 h. (B) Annexin-PI assay assessed the percent of apoptotic cells by flow cytometry, as described in the Materials and Methodology. (C) NO production by THP-1 cells upon infection with *M.s*_ ThyX and *M.s*_Vc for 24 and 48 h. LPS (100 ng/mL) was used as the positive control. Data were plotted as NO concentrations (in micromolar). The treated and untreated groups were statistically compared. All statistical analyses were performed using two-way ANOVA. The *P* values for *, **, ***, and ns are <0.05, < 0.01, < 0.001, and >0.05, respectively.

By halting the release of intracellular pathogens and the propagation of mycobacterial infection, apoptosis is essential to the host’s defense against intracellular infections, such as *M.tb*.^15^ Innate and adaptive immune responses are triggered by macrophage apoptosis, which can reduce mycobacterial infection.^16^ Apoptotic bodies that contain bacteria, other cellular organelles, and cell cytoplasm are picked up by dendritic cells and macrophages by receptor-mediated phagocytosis.^17^ An early stage of the infection is usually eliminated by apoptosis, a programmed cell death that protects the host cells. However, it can favor the bacterium in the later stages of infection by disseminating the disease via apoptotic bodies.^18^ Accordingly, we investigated the effect of ThyX in macrophages infected with *M.s*_ThyX and *M.s*_Vc. The recombinant strains’ potential to cause apoptosis was examined by checking apoptotic markers using flow cytometry analysis (Figure 2A). We have analyzed apoptosis 48 h postinfection and observed that overexpression of ThyX effectively enhances the apoptosis in infected macrophages compared to the vector control (Figure 2B).

Free radicals play an important role in controlling bacterial infection.^19^ Hence next, we checked the levels of NO in *M.s*_ThyX- and *M.s*_Vc-infected cells. Interestingly we have found increased NO levels in *M.s*_ThyX-infected cells compared to the vector control (Figure 2C). These results together suggest that overexpression of ThyX stimulates different host defense mechanisms and indicate that the *M.s*_ThyX provides the capability of nonpathogenic bacterium to stimulate NO production from host macrophages, followed by macrophage cell death by apoptosis.

### 2.4 ThyX Confers Resistance to Oxidative and Hypoxic Stress Conditions

**Figure 3:**
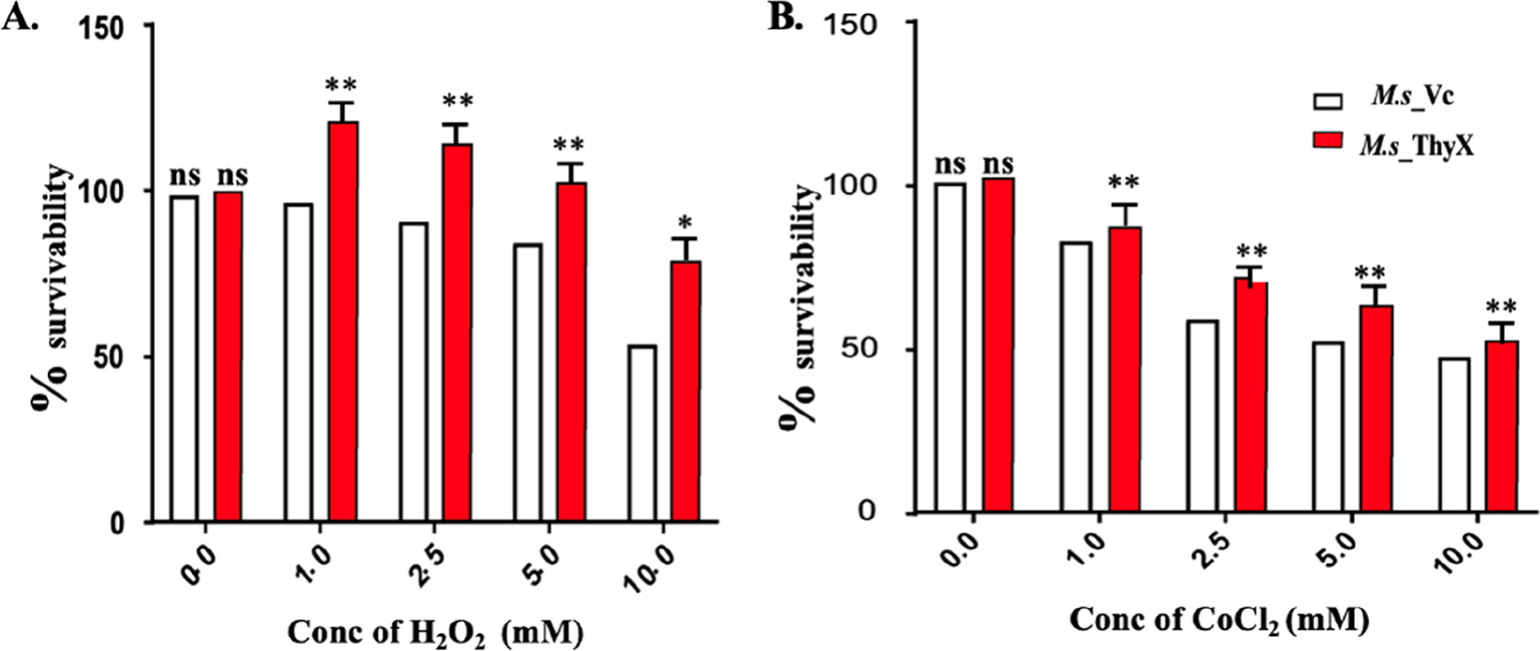
*Mycobacterium tuberculosis* ThyX protects the bacteria against oxidative and hypoxic stress conditions. Recombinant *M.s*_Vc (white bars) and *M.s*_ThyX bacterial cells (red bars) were grown in the presence of oxidative (H_2_O_2_) (A) and hypoxic (CoCl_2_) (B) stress environments. Cell viability was assessed using 0.3% resazurin sodium salt for 4 h spectrophotometrically. Data were plotted as percent survivability.

An infection spreads as apoptotic bodies transfer bacteria to nearby cells. The hypoxic and acidic environments created by infected macrophages kill the mycobacteria. *M.tb* provides a novel means of survival in macrophages by establishing a tolerance to acidic and hypoxic stress environments. Many proteins secreted by *M.tb* can provide defense against oxidative and hypoxic stress. It is known that H_2_O_2_ and CoCl_2_ can induce oxidative stress and hypoxic stress, respectively. Here, we were interested in checking whether ThyX overexpression affects bacterial survival under oxidative and hypoxic stress conditions. Bacterial cells of both strains were seeded independently in a concentration-dependent manner. The oxidative stress was generated through the addition of H_2_O_2_ in different concentrations ranging from 1 to 10 mM, and survival of bacteria was checked after 24 h through the Alamar blue assay. Here we have observed a better survival of *M*.s_ThyX compared to *M*.s_Vc (Figure 3A). Similar results were observed in the case of hypoxic stress induced by the addition of CoCl2 (1 to 10 mM) as well (Figure 3B).

### 2.5 *M*.*tb* ThyX Upregulates Macrophage Activation

**Figure 4:**
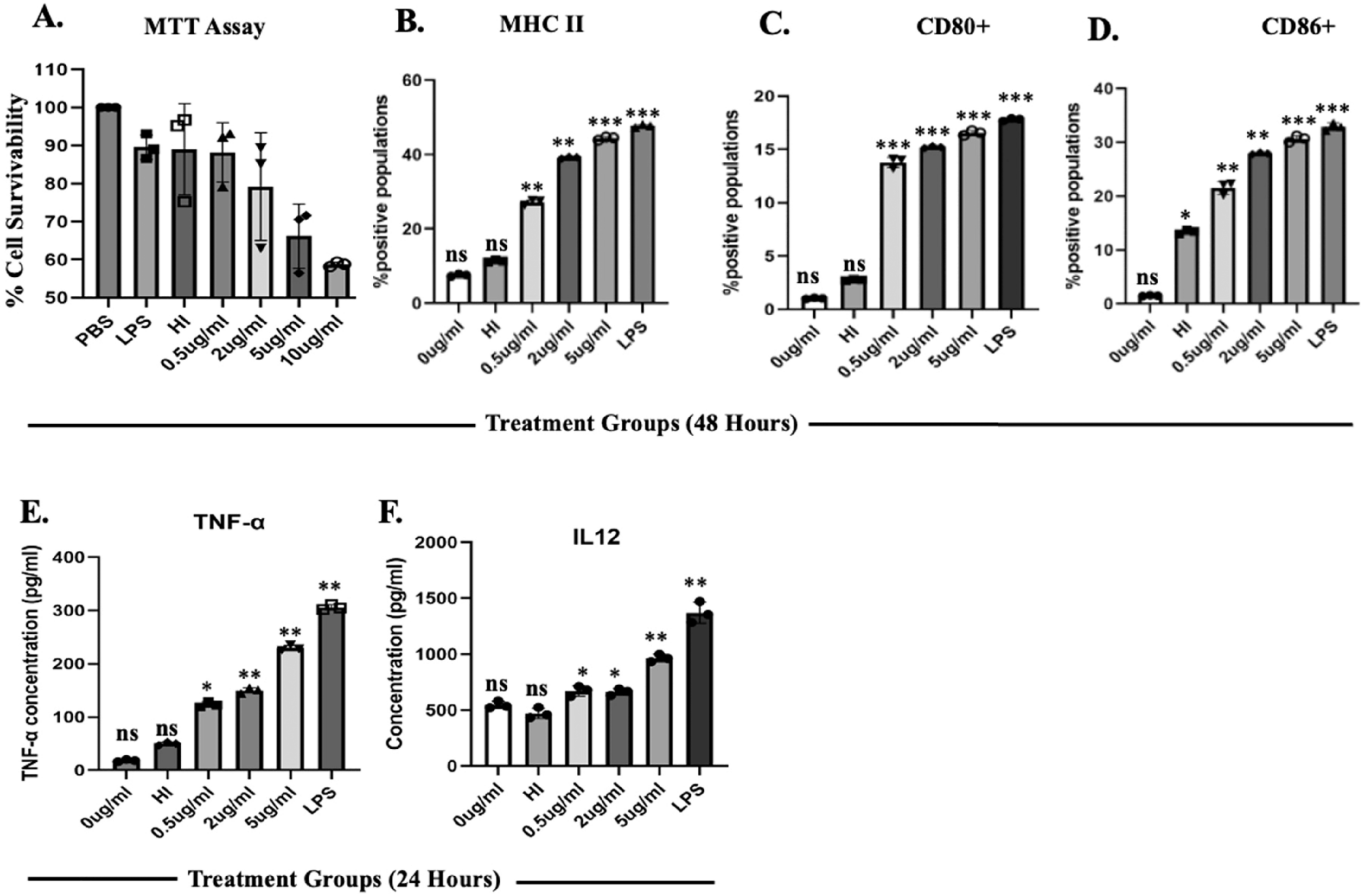
RAW264.7 cells were treated with (A) ThyX, and cell viability was assessed spectrophotometrically through the MTT assay. (B) ThyX enhances the expression of macrophage activation markers. Quantitative representation of the expression of (B) MHCII, (C) CD80, and (D) CD86 on the surface at 48 h. (E) Culture supernatants were collected at 24 h postinfection, and the concentrations of TNF-α (E) and IL-12 (F) were determined using ELISA. Representative data from three experiments show the concentration of TNF-α and IL-12. Representative data obtained from three independent experiments show means ± SEM of duplicate wells. *P* value of <0.01.

A functional CD4+ T-cell response depends on the controlled expression of CD80/CD86 and MHC-II (major histocompatibility complex). Effective T-cell activation and cytokine generation are achieved by co-stimulatory signal molecules, CD80, CD86, and macrophage activation marker MHCII.^20,21^ As we have observed in the above results, ThyX shows antigenic properties; hence, we have checked the expression of macrophage activation markers in the presence of ThyX. To do that, we performed *ex-vivo* experiments using RAW264.7 macrophages. Initially, cells were exposed to different concentrations (0.5 to 5 μg/mL) of ThyX purified protein along with lipopolysaccharide (LPS) as a positive control and heat-inactivated (HI) protein as a negative control. First, we checked the survival of cells through the MTT assay and found there was no significant cell death until 5 μg/mL ThyX (Figure 4A). Hence for further experiments we have used protein concentration in a range. At 48 h, fluorescence-activated cell sorting (FACS) analysis was performed to check the levels of different macrophage activation markers and showed that increasing concentrations of ThyX significantly enhance the expression of CD80, CD86, and MHC II (Figure 4B, C, and D). It is possible that *M.tb* ThyX modulates T cell activity through an increase in the expression of MHC II, CD80, and CD86.

Macrophage activation leads to T-cell activation and the generation of different protective cytokines to clear the infection. Therefore, we were interested in checking whether ThyX protein is involved in the stimulation of proinflammatory cytokines. Here, we have observed upregulated levels of IL-12 and TNFα in the presence of ThyX protein (Figure 4E and F).

### 2.6 Exposure to *M. tb* ThyX *in Vivo* Also Causes Apoptosis and Increases NO and ROS

To eradicate infection, macrophages create increased amounts of ROS and NO.^22^ If they cannot eradicate the pathogen, they may also undergo apoptosis.^23^ These findings offer a molecular explanation for the activation of apoptosis in macrophages harboring ThyX. Virulence and cell death that bacteria use to cause disease are indeed correlated.^24^

**Figure 5:**
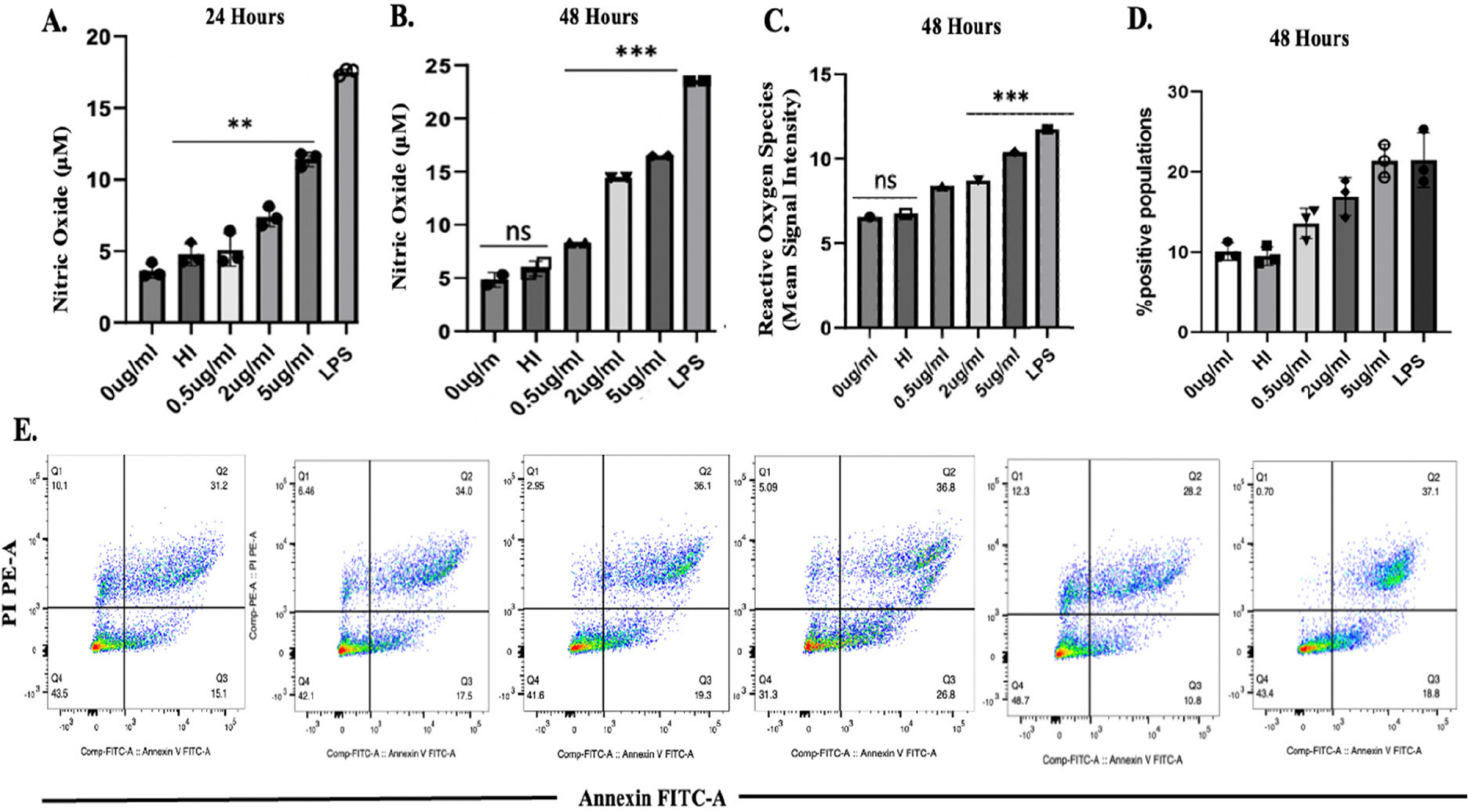
THP-1 cells were cultured in the absence or presence of ThyX. NO production by THP-1 cells upon infection with recombinant ThyX for (A) 24 and (B) 48 h. Data were plotted as the NO concentrations (μM). (C) ROS level was analyzed by flow cytometry. The mean fluorescent intensity (MFI) of intracellular ROS production in infected macrophages was measured at 48 h. (D) Annexin-PI assay assessed percent apoptotic cells by flow cytometry, as described in the Materials and Methodology. (E) Representative bar graph of an apoptosis assay at 48 h. LPS (100 ng/mL) was used as the positive control.

To assess the role of ThyX’s significance in the protection of the bacteria residing within the macrophages, THP-1 cells were treated with purified ThyX. The macrophage cells treated with ThyX were seen to have elevated NO levels (Figure 5A and B). ROS level was quantified by flow cytometry using the CellROX Green Reagent assay. The figure indicates that the ROS level increases with an increase in the concentration of ThyX in treated macrophages at 48 h. It was observed that ThyX-treated macrophages produced significantly higher levels of ROS with increasing concentrations of protein (Figure 5C). The ThyX gene helps the pathogen increase the levels of ROS produced by the host, which allows the bacteria to live inside macrophages.

When infected macrophages undergo apoptosis, innate control over early bacterial growth is established. Additionally, the antigen-containing reservoir serves as a bridge for dendritic cells to initiate acquired T-cell immunity.^25,24^ Apoptosis serves as a last-resort host defense mechanism. Controlled bacterial survival is accomplished by enclosing pathogens within apoptotic cells.^26^ Additionally, bacterial antigens that can activate *M.tb*-specific T-cell immunity are largely obtained from apoptotic macrophages.^27^ However, a growing body of evidence indicates that pathogenic *M.tb* generates bacterial chemicals that prevent apoptosis and instead cause necrosis in macrophages.^28^ The percent apoptotic cells was estimated after 48 h using a FITC Annexin V apoptosis detection kit. We observed a significant increase in apoptosis in macrophages infected with ThyX in a concentration-dependent manner in the late apoptotic phase (Figure 5D and E).

## 3 Conclusion

In this research, we delve into the multifaceted role of the *M.tb* ThyX protein in the context of tuberculosis infection. The study investigates how ThyX influences the delicate interplay between the host’s immune response and the pathogen’s survival strategies.

Our findings reveal a complex picture. *M.tb* ThyX modulates the antigen processing pathway, stimulating macrophages and enhancing the expression of co-stimulatory markers on antigen-presenting cells. This increased expression of MHC II, CD80, and CD86 molecules on macrophages treated with ThyX suggests a role in enhanced antigen presentation, impacting immune responses and pathogen clearance, potentially hindering their ability to recognize and eliminate the invading *M.tb* bacteria. ThyX appears to trigger the release of pro-inflammatory cytokines such as TNF-α and IL-12, enhancing the host’s immune responses against *M.tb* and potentially bolstering the host’s immune defenses.

Furthermore, the results suggest that ThyX may equip *M.tb* with an enhanced resistance to the harsh environment within macrophages. This includes increased tolerance to stressors like reactive oxygen species (ROS) and hypoxia, conditions typically employed by macrophages to combat bacterial threats. ThyX induces stress responses in macrophages, leading to the upregulation of ROS production, creating an unfavorable environment for the pathogen. Moreover, ThyX promotes NO generation from host macrophages, leading to apoptosis-induced macrophage cell death and potentially limiting bacterial multiplication.

ThyX contributes to the resistance of *M.tb* to macrophage stress conditions, allowing the bacteria to survive and grow in hostile environments. Our results have shown that *M. smegmatis* strains overexpressing ThyX exhibit higher survival rates and persistence within macrophages than control strains. This ability of ThyX to enhance bacterial survival and persistence underscores its significance in *M.tb* pathogenesis.

In conclusion, this study sheds light on the multifaceted role of *M.tb* ThyX in TB pathogenesis by influencing immune modulation, antigen presentation, cytokine secretion, and bacterial survival within host macrophages. While it may activate the host’s immune response, it also equips *M.tb* with mechanisms to evade and potentially manipulate these defenses. Considering these findings, targeting ThyX holds the potential for developing novel therapeutic strategies to combat TB. However, further research is needed to fully understand the complex interplay between ThyX and the host immune response.

## 4 Materials and Methodology

### 4.1 Reagents, Chemicals, Vectors, and Bacterial Strains

Analytical grade reagents and chemicals were used in this study. The cell culture growth media (DMEM and RPMI), antibiotic (antibiotic–antimycotic), and fetal bovine serum (FBS) were purchased from Gibco Life Technologies. Hi-Media Laboratories (India) supplied the LB broth used to grow the bacterial culture. The PCR reagents were purchased from Fermentas (Thermo Fisher Scientific, Inc., CA, USA). A gel extraction and plasmid isolation kit was purchased from Qiagen. The gene-specific primers were purchased from Sigma. *M. smegmatis* mc2155 was borrowed from the National Institute of Pathology (NIOP), India. The sequence of genes was retrieved from Mycobrowser. Both strains were grown in LB broth medium at 37 °C under continuous shaking at 180 rpm. Kanamycin was added at a final concentration of 50 μg/mL.

### 4.2 Molecular Cloning, Expression, and Purification of ThyX

To produce the ThyX protein, *Escherichia coli* Rosetta cells were used to express the ThyX protein after the ThyX gene was PCR amplified and cloned into a pET28a expression vector. For protein purification, 30 mL of chilled 1× PBS (pH 7.4) was used to resuspend the IPTG-induced culture pellet, and 150 mM KCl was mixed properly and kept on ice for 5 min. For 5 to 10 min, the cell suspension was sonicated with an amplitude of 40% with regular off and on cycles of 10 and 5 s each, respectively. The sonicated product was centrifuged at 9000 rpm for 45 min, and the supernatant containing the solubilized protein was collected and loaded on the Ni-NTA (Qiagen/Genetix) column. Further washing was done with 50 mL of 40 mM imidazole in 1× PBS. Protein was eluted with a 200 mM imidazole-containing buffer. Fractions containing recombinant protein were analyzed on 15% SDS-PAGE. The Bradford assay was used to determine the concentration of the dialyzed proteins. Polymyxin B (Sigma) was added to the protein at 4 °C for 2 h for removal of lipopolysaccharides.

### 4.3 Cloning of *M.tb* ThyX in Mycobacterial Expression Vector pVV16 and Generation of Recombinant *M. smegmatis*

The ThyX encoding gene was subcloned into mycobacterial integration expression vector pVV16 to produce the pVV16_ThyX plasmid.^29^ This construct along with empty vector pVV16 was electroporated (Bio-Rad Laboratories, CA, USA) into competent *M. smegmatis* to generate recombinant strains termed *M.s*_ThyX and *M.s*_Vc. To confirm the expression of recombinant ThyX, for 24 h, *M.s_*ThyX and *M.s*_Vc were grown in LB broth that was supplemented with 50 μg/mL kanamycin. Centrifugation was used to obtain the cell pellet, which was then PBS-washed (5000 rpm, 10 min). The cell pellet was heated at 95 °C for 30 min after being dissolved in SDS-PAGE loading dye. ThyX protein expression was confirmed by Western blotting with an anti-His antibody after the lysed fractions were separated by electrophoresis in 10% SDS-PAGE.

### 4.4 Macrophage Cell Culture and Growth Conditions

The human macrophage cell line THP-1 and murine macrophage RAW264.7 cells were cultured in Dulbecco’s modified Eagle’s medium (DMEM) and Roswell Park Memorial Institute (RPMI 1640) supplemented respectively with 1% antibiotic solution and 10% FBS. Depending on the experiment, the necessary number of cells was seeded in six-well and 96-well plates. The cells were treated with various concentrations of recombinant ThyX protein or with *M.s*_ThyX and *M.s*_Vc. Under standard tissue culture conditions of 37 °C and 5% CO_2_, cells were grown and maintained.^30^ After the initial frozen stocks were seeded, eight passages later, all experiments were completed in the different cell lines.^30^

### 4.5 *In-Vitro* Survival of *M.s*_Vc and *M.s*_ThyX under Normal Growth Conditions

*M. smegmatis* mc2 155 vector control (*M.s*_Vc) and *M.s*_ThyX log phase cultures (OD_600_ of 0.8–1.0) were grown for about 12 h until the OD_600_ reached 0.05 after being diluted 1:100 onto LB medium.^30^ The cells were reinoculated and were allowed to grow for 30 h, and the OD_600_ was taken after every 3 h up to 30 h.

### 4.6 *In-Vitro* Stress Response Assay

*M.s*_ThyX and *M.s*_Vc were raised to an OD of 1.0 and further diluted to an OD of 0.2 in fresh LB media. After that, the bacterial cells were seeded into 96-well plates and given 24 h to develop. After, a 24 h growth period, 1–10 mM H_2_O_2_ and 1–10 mM CoCl_2_ were used to induce oxidative and hypoxic stress, respectively. With 0.3% resazurin sodium salt, cell viability was evaluated after 24 h by monitoring the readings at 570 and 600 nm in a spectrophotometer and calculating the survival percentage.

### 4.7 Bacterial Survivability Assessment in Infected Macrophages

Recombinant *M. smegmatis* expressing ThyX was added to PMA-differentiated THP-1 macrophages along with the vector control grown to an OD of 0.1 at an MOI (multiplicity of infection) of 1:10 in the BSL2 facility.^30^ The macrophages were lysed and serially diluted after 0, 24, 48, and 72 h and then plated on Luria agar plates to allow the bacterial colonies to develop. After respective hours of incubation at 37 °C, the CFUs of the bacterial colonies were counted to determine the viability of the bacteria.

### 4.8 MTT Assay

The MTT assay was done to check the cytotoxicity of *M.tb* protein ThyX. The assay was carried out using RAW264.7 cells (1 × 104/well) seeded in 96-well plates in complete DMEM media and treated with proteins in different concentrations for 24 h. A fresh 200 μL medium was added once the supernatant was harvested. MTT was diluted to a final volume of 20 μL and incubated at 37 °C for 4 h. After completely removing the media, 100 μL of DMSO was added to each well, and it was thoroughly mixed. Absorbance was measured at 595 nm.

### 4.9 Surface Expression of Macrophage Activation

Various concentration of ThyX protein (0.5, 2, and 5 μg/mL) were added to macrophage RAW264.7 cells, and the surface activation markers for macrophage activation, such as MHC-II, CD80, and CD86, were determined. Cells in 24-well culture plates were seeded and treated with recombinant ThyX protein after 4 h of seeding. They were then incubated for 48 h with anti-mouse Alexa Fluor 488-MHCII, PE-CD80, and APC-CD86. The samples were handled by the supplier’s supplied protocol. LPS (100 ng/mL) was used as a positive control for the expression of TLR4.

### 4.10 Estimation of Cytokine Levels

In a 12-well culture plate, murine macrophage cells were seeded (∼1 × 10^6^ cells per well), and the cells were left to adhere overnight at 37 °C. After adhesion, cells were treated with recombinant ThyX protein at varying concentrations (0.5, 2, and 5 μg/mL) or with LPS as a positive control (100 ng/mL) (Sigma, USA). To release cytokines and other cellular markers, the concentration of the protein treatment was prestandardized. Heat-inactivated proteins are used as a negative control for cytokine estimation.^30^ After 24 h of treatment, the supernatant was removed and stored at −80 °C until required. Pro-inflammatory cytokines, such as TNF-α and IL-12, and anti-inflammatory cytokines, IL-10, were measured at 450 nm using the BD Biosciences mouse ELISA kit by following the manufacturer’s instructions.

### 4.11 Detection of ROS in Macrophages

THP-1 cells (2 × 10^5^ cells/well) were seeded overnight and were treated with recombinant ThyX at concentration ranges from 0.5, 2, and 5 μg/mL at 37 °C in a 12-well plate. Cells were collected and washed with 1× PBS after 48 h of treatment. About 5 mM CellROX green reagent was used to stain the treated cells, followed by incubation at 37 °C for 30 min. Stained cells were captured using FACS Lyric (BD Biosciences), and Flow Jo software (Tree Star) was used to analyze the data.

### 4.12 Quantification of Nitrite (NO) in Macrophages

The THP-1 cells were treated with recombinant strains that expressed ThyX, *M.s_Vc*, and *M.s*_ThyX. Around 150 μL of the cell-free supernatant was collected after 30 h of treatment and was mixed with a volume of 50 μL of Griess reagent. This reaction was carried out in 96-well plates and incubated at room temperature for 30 min at 37 °C, 5% CO_2_. Untreated macrophage cells were used as control. The cells were harvested 24 and 48 h after infection. Nitrite concentration was measured using sodium nitrite as a standard. Plates were measured at 540 nm.

### 4.13 Annexin V/PI Apoptosis Assay

THP-1 cells seeded in 24-well plates were incubated with 0.5, 2, and 5 μg/mL of recombinant ThyX protein. THP-1 cells were also treated with *M.s*_Vc and *M.s*_ThyX at an MOI of 1:10 and were incubated for 4 h. Post-treatment, to kill the extracellular bacteria, cells were treated with complete media containing gentamycin after being washed with PBS^30^ in the case of the *M. smegmatis* strain. In both cases, after treatment, cells were harvested after 48 h and stained with AnnexinV-FITC and propidium iodide staining protocol (BD) to analyze apoptosis. Cells were washed and collected in PBS and then resuspended in 1× binding buffer. The treated cells, approximately 1 × 10^5^, were transferred into fresh tubes, and 5 μL of FITC AnnexinV and PI were added to each. Cells were gently vortexed and incubated at RT for 15 min. Following the addition of 400 μL of binding buffer to each tube, cells were examined using flow cytometry at the correct machine settings. The positive control was performed using LPS-treated cells. FACS Lyric (BD Biosciences, San Jose, CA, USA) was used to analyze the samples, and Flow Jo software was used to process the data.

### 4.14 Statistical Analysis

GraphPad Prism 6.0 software was used to express all data, which were obtained from three independent groups of experiments and expressed as mean ± standard deviation (SD). A one-way analysis of variance (ANOVA) was used to determine the statistical significance at *p* < 0.05.

## Supporting Information Available

Figure S1: Online tools used to predict antigenicity, immunogenicity, and B-cell and T-cell epitopes of ThyX; Figure S2: Cloning, expression, and purification of ThyX (Rv2754c) (PDF)
