# Supplementary material for: Mycobacterium tuberculosis Essential Gene Thymidylate Synthase Is Involved in Immune Modulation and Survival inside the Host
Source: ACS Omega. 2024 Jul 23;9(31):33743–50. doi: 10.1021/acsomega.4c02919 (PMC11308015; doi:10.1021/acsomega.4c02919)
Supplement: Supplementary file 1 — ao4c02919_si_001.pdf [file ao4c02919_si_001.pdf]

Mycobacterium Tuberculosis essential gene Thymidylate synthase is involved in immune modulation and survival inside the host

Sana Tanweer[1#], Tarina Sharma[2], Abhinav Grover[3], Meetu Agarwal[1*], Sonam Grover[1*]

[1] *Department of Molecular Medicine, Jamia Hamdard, New Delhi-110065*

[2] *New Jersey Medical School, Rutgers, The State University of New Jersey, Newark, New Jersey 07103, United States*

[3] *School of Biotechnology, Jawaharlal University, New Delhi-110069*

***To whom the correspondence should be addressed**

E-mail address: sonamgbt@gmail.com, meetuagarwal2388@gmail.com

Supplementary Figure

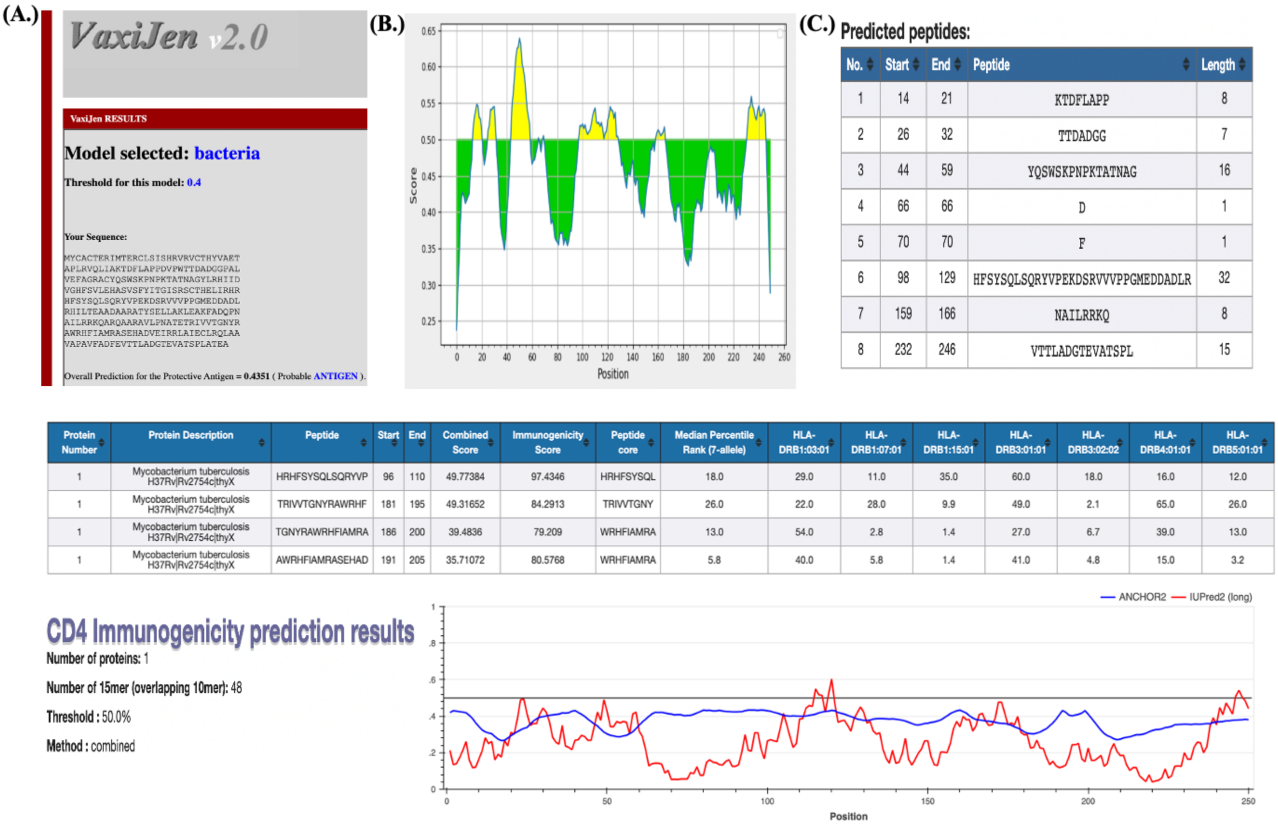

No.	Start	End	Peptide	Length
1	14	21	KTDFLAPP	8
2	26	32	TTDADGG	7
3	44	59	YQSWSKPNPKTATNAG	16
4	66	66	D	1
5	70	70	F	1
6	98	129	HFSYSQLSQRYVPEKDSRVVVPPGMEDDADLR	32
7	159	166	NAILRRKQ	8
8	232	246	VTTLADGTEVATSPL	15

Protein Number	Protein Description	Peptide	Start	End	Combined Score	Immunogenicity Score	Peptide core	Median Percentile Rank (7-allele)	HLA-DRB1:03:01	HLA-DRB1:07:01	HLA-DRB1:15:01	HLA-DRB3:01:01	HLA-DRB3:02:02	HLA-DRB4:01:01	HLA-DRB5:01:01
1	Mycobacterium tuberculosis H37Rv|Rv2754c|thyX	HRHFSYSQLSQRYVP	96	110	49.77384	97.4346	HRHFSYSQL	18.0	29.0	11.0	35.0	60.0	18.0	16.0	12.0
1	Mycobacterium tuberculosis H37Rv|Rv2754c|thyX	TRIVVTGNYRAWRHF	181	195	49.31652	84.2913	TRIVVTGNY	26.0	22.0	28.0	9.9	49.0	2.1	65.0	26.0
1	Mycobacterium tuberculosis H37Rv|Rv2754c|thyX	TGNYRAWRHFIAMRA	186	200	39.4836	79.209	WRHFIAMRA	13.0	54.0	2.8	1.4	27.0	6.7	39.0	13.0
1	Mycobacterium tuberculosis H37Rv|Rv2754c|thyX	AWRHFIAMRASEHAD	191	205	35.71072	80.5768	WRHFIAMRA	5.8	40.0	5.8	1.4	41.0	4.8	15.0	3.2

Figure S1: (A)Antigenicity was predicted by the VaxiJen tool (https://www.ddg-pharmfac.net/vaxijen/VaxiJen/VaxiJen.html). (B) *In-silico* immune analysis of ThyX protein B-cell epitopes prediction using IEDB tool. The regions in yellow are B-cell epitopes above the threshold limit. (C) Predicted peptides are the Number of B-cell epitopes above the threshold limit. (D) T-cell epitope prediction in ThyX by IEDB tool (http://tools.immuneepitope.org/bcell).

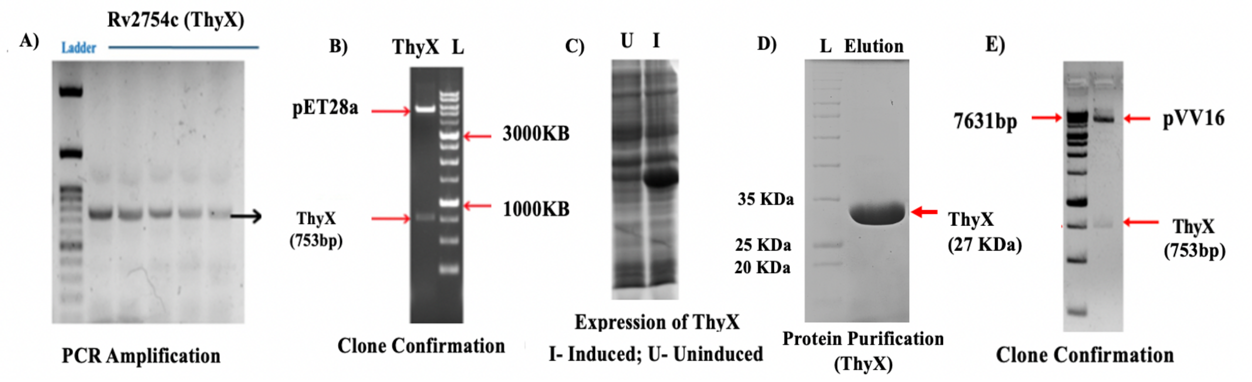

Figure S2: Expression and construction of ThyX` in E.coli (A) PCR amplification of ThyX from *M.tb* gDNA (Lane 1: Marker, Lane 2-6: amplicon). (B) Cloning of ThyX in expression clone pET28a vector and restriction digestion for confirmation of clone. (Lane 1: digested products, Lane 2: Marker). (C) expression of ThyX, induction was done by IPTG (Lane 1; uninduced, Lane2; Induced culture). (D) SDS-PAGE image depicting the elution of recombinant *M.tb* ThyX. A distinct band is observed at 27kDa (Lane 1; Ladder, Lane 2; Elution). (E) Cloning of ThyX in expression clone pVV16 vector and restriction digestion for confirmation of clone. (Lane 1: Marker, Lane 2: digested products).
